# The effect of anaesthesia on flank incisional pain: infiltration versus intercostal nerve block, a comparative study

**DOI:** 10.11604/pamj.2020.36.356.24279

**Published:** 2020-08-27

**Authors:** Awad Al-Kaabneh, Adnan Abo Qamar, Firas Al-Hammouri, Ashraf Al-Majali, Ali Alasmar, Nizar Al-sayedeh, Firas Khori, Anan Qapaha, Mohammad Beidas

**Affiliations:** 1 Department of Urology, Prince Hussein Urology Institute, King Hussein Medical Centre, Amman, Jordan,; 2 Anaesthesia Department, King Hussein Medical Centre, Amman, Jordan

**Keywords:** Flank incision, local anaesthesia, wound infiltration, intercostal nerve block

## Abstract

The object of this study is to determine which local wound analgesic option is superior, local anaesthetic infiltration or intercostal nerve block, by combined local anaesthetic agents (0.5% bupivacaine + 2% lidocaine) and to detect which option can best alleviate the post-operative pain management and significantly prolong the time to the first rescue analgesic requirement and the total consumption of opioids in the first post-operative 72 hrs. The medical records of 1458 patients who underwent flank incision procedures by two different surgeons in our institute were retrospectively reviewed. Each surgeon used a different type of local incisional pain management; the first one used infiltration of flank incision routinely, the second surgeon used an intercostal block with all his patients. These elective procedures were carried out in our Urology Centre between June 2007 and June 2019. The duration of follow-up was from the recovery transfer until the end of the third post-operative day. Patients were divided into two groups: group 1 (729 patients-infiltration of flank incision) and group 2 (729 patients-intercostal nerve block). Patients were aged between 19-78 years. No significant differences were seen regarding the demographic data between both groups, P > 0.05. On the other hand, there were significant differences between group 1 and group 2 according to the mean visual analogue scale score (lower in group 1, P < 0.05), the total mean analgesic requirements during the first post-operative 72 hrs (lower in group 1, P < 0.05) and the time to the first analgesic demand (higher in group 1, P < 0.05). There were no statistically significant differences in post-operative complications between both groups, P > 0.05. The infiltration of flank incision with combined local anaesthetic agents (0.5% Bupivacaine + 2% lidocaine) is more effective in alleviating post-operative pain, decreasing total analgesic consumption during the first post-operative 72 hrs and prolonging the time required for the first rescue opioid.

## Introduction

In open renal surgery, adequate exposure of the field must be achieved to complete the procedure well and to handle any possible complications. Thus, the need for large incisions is mandatory in renal surgery because small incisions could lead to poor exposure and difficulty in controlling injuries to the renal vascular pedicle especially when a large tumour is present. The flank approach in renal surgery provides excellent exposure of the renal parenchyma and collecting system, and the most commonly used flank approach is through the bed of the 11^th^ or 12^th^ rib [1]. The most common indications of flank incision in renal surgery are nephrectomy (partial vs. complete renal excision, and simple vs. radical), renal parenchymal cyst excision, open nephrolithotomy, nephroureterectomy, ureteroureterostomy and open pyeloplasty for pelvi-ureteric junction obstruction [2]. Renal surgery, especially the open method, carries a risk of several complications (1-immediate, like vascular, splenic and bowel injury; 2-early, like acute renal failure, bowel obstruction, peritonitis, deep venous thrombosis (DVT) and pulmonary embolus; 3-late, like chronic renal failure, incisional hernias and wound infection). Also, flank incision plays a role in these complications by increasing the post-operative pain, and consequently delaying patient recovery, and increasing hospital stay which causes ileus, increases opioid demand and decreases patient mobility from bed with resultant DVT or pulmonary embolus, besides weakening the abdominal wall due to the damage of muscles and nerves which later causes incisional hernia [3]. Therefore, despite the fact that flank incision has been used by urologists for decades, its morbidity has always been an issue [4].

Opioids are the cornerstone of post-operative pain control; therefore, one of the most important roles of the anaesthesiologist is to achieve effective and adequate post-operative analgesia to provide good post-operative pain management and early ambulation with minimal complications [5]. Enhancing post-operative pain depends on multiple factors: surgical (procedure type, length and approach), patient response to pain and surgery and pre/peri-operative analgesic methods. Thus, to meet early patient discharge criteria, post-operative pain and side effects (nausea, vomiting and ileus) should be controlled. One of the options that meets this criterion is limiting the requirement for opioid analgesics by local anaesthetic wound infiltration [6]. The purpose of this study was to investigate the efficacy of flank incision infiltration by local anaesthetic agents such as 0.25% Bupivacaine on post-operative pain control and compare it with intercostal nerve block by the same agent to clarify which method was the most effective in pain management, decreasing the requirements of total analgesic consumption in 24 hrs and prolonging the time to first rescue analgesic demand.

## Methods

In total, 1458 patients with flank incision elective procedures were enrolled in the study. The procedures performed on these patients were nephrectomy (partial vs. complete and simple vs. radical), open nephrolithotomy, pyeloplasty, nephroureterectomy and ureteroureterostomy, and procedures were performed between June 2007 and June 2019 in our urology centre. The procedures were carried out by two surgeons, one who used infiltration of flank incision routinely and the second who used intercostal block for all his patients. Retrospectively, we reviewed the medical records of these patients and, with the help of our assistants, we divided the records into two groups: group 1-729 patients, incisional infiltration (ICI) of combined local anaesthetic agents (0.5% bupivacaine (20ml) + 2% lidocaine (5ml) diluted in 15ml 0.9% normal saline solution) intra-operatively; and group 2-729 patients, intercostal nerve block (ICNB) with the same anaesthetic agent, also intra-operatively. The patients ranged from 19 to 78 years in age, and the follow-up period of each patients was from the day of surgery, post-operatively in the recovery room after the patient became fully conscious, until the end of the third post-operative day.

Patient´s inclusion criteria included renal pathologies (benign or malignant tumours, benign or malignant cysts, stones, non-functioning kidney and chronic infections (granulomatous pyelonephritis); ureteral pathologies (pelvi-ureteric junction obstruction, upper ureteral tumours and elective repair of ureteral injuries); donor nephrectomy; and controlled bleeding profile, diabetes mellitus (DM) and hypertension (HTN). On the other hand, exclusion criteria were ages < 19 or > 78 years; patients on dialysis; pregnancy; ectopic kidneys; uncontrolled haematuria by selective angioembolization; renal injuries that needed nephrectomy; and acute infections (abscesses or emphysematous pyelonephritis). All patients were given third generation cephalosporins pre-operatively (ceftriaxone 1 gr. I.V.), and blood samples with urine cultures were confirmed to be normal pre-operatively. Also, a double J catheter (DJC) was inserted pre-operatively if required.

The procedures were performed through retroperitoneal flank incision providing the whole known steps of open renal surgery. The level of incision was subcostal, supracostal or transcostal (with excision of 11^th^ or 12^th^ rib, in general). The decision on which type of incision depended on the position of the kidney and the exposure required. The most common flank approach used was supracostal incision-supra twelfth rib incision. The local anaesthesia technique was as follows: at the end of the surgery, ICI with 40ml of diluted combined local anaesthetic agents (20ml of 0.5% bupivacaine + 5ml of 2% lidocaine + 15ml 0.9% NaCl) in group 1 and ICNB of the same diluted combined agents at the termination of the procedure in group 2 were applied.

**Statistical analysis:** categorical data are expressed in frequency and percentages, while scale data is expressed in mean and standard deviation (SD). Chi square of independence was used to find associations between categorical data, split plot ANOVA was used for group mean differences across all observation intervals. An alpha level of ≤ 0.05 was considered statistically significant with power of study 80. SPSS version 25 was used to analyse the data.

## Results

The patients were aged from 19-78 years, with group 1 patients aged between 21-78 years and group 2 patients between 19-75 years, and the mean ± SD values being 49.5 ± 13.88 and 48.6 ± 16.47 years for group 1 and group 2, respectively. Of the 1458 patients, 806 were males and 652 females, and there were more left-sided nephrectomies than right-sided nephrectomies (753 in the left side and 705 in the right side). The demographic data of all patients is presented in Table 1, together with the percentages of each group variable, calculated in relation to the total number of patients (Table 2). A significant P value was considered < 0.05. VAS^*^: visual analogue scale. IFI^®^: Infiltration of Flank Incision. ICNB^®^: InterCostal Nerve Block. SD➊: Standard deviation. TAR➋: Total analgesic requirements.

**Table 1:** demographic data of the study patients

Variables (n^*^/%)	Group 1 (IFI^®^)	Group 2 (ICNB^®^)	P value
**Ages**			
19-40 years	(187/13%)	(193/13.2%)	0.720
41-60 years	(356/24%)	(344/23.6%)	0.529
61-78 years	(186/13%)	(192/13.2%)	0.720
**Gender**			
Males	(401/27.5%)	(405/27.8%)	0.833
Females	(328/22.5%)	(324/22.2%)	0.833
**Laterality**			
Left	(372/25.5%)	(381/26%)	0.637
Right	(357/24.5%)	(348/24%)	0.637

n^*^: number of patients. IFI^®^: Infiltration of Flank Incision. ICNB^®^: InterCostal Nerve Block.

**Table 2:** differences between groups regarding the mean visual analogue scale (VAS) score

Variables	Group1 (IFI^®^)	Group 2(ICNB^®^)	P value
Mean VAS score^*^ ± SD➊			
6 hrs	5.56 ± 1.01	6.73 ± 1.05	≤0.001
12 hrs	4.32 ± 0.852	5.95 ± 1.15	≤0.001
24 hrs	3.11 ± 0.92	4.36 ±0.752	≤0.001
48 hrs	2.27 ± 1.23	3.65 ± 1.096	≤0.001
72 hrs	1.83 ± 0.064	2.87 ± 1.025	≤0.001
TAR➋ (mean ± SD) (mg of tramadol intramuscular injection IM)	166.049 ± 107.878	235.288 ± 118.235	≤0.001
The time to first analgesic demand (mean ± SD) (hrs)	4.65 ± 1.65	2.987 ± 1.51	≤0.001
Mean changes in haematocrit (%) ± SD	5.151 ± 1.673	5. 23 ± 1.741	0.377
Mean blood transfusion rate (U) ± SD	4.21 ± 2.762	4.071 ± 2.914	0.350
Mean hospital stay (days) ± SD	5.901 ± 1.054	5.82 ± 1.107	0.152
Complications (%)			0.0972
Mean Clavien I score	8.275	6.35	
Mean Clavien II score	14.725	11.15	
Mean Clavien IIIA score	3.5	3.725	
Mean Clavien IIIB score	2.125	1.925	
Mean Clavien IVA score	0	0	
Mean Clavien IVB score	0	0	
Mean Clavien V score	0	0	
Mean Clavien score (mean complications rate)	28.625	23.15	

VAS^*^: visual analogue scale. IFI^®^: Infiltration of Flank Incision. ICNB^®^: InterCostal Nerve Block. SD➊: Standard deviation. TAR➋: Total analgesic requirements

## Discussion

Traditional open nephrectomy exposes the patient to a large flank incision [7], which prolongs the patient´s recovery due to significant post-operative pain [8], besides which those patients may experience chronic post-operative pain [9]. In urological procedures, the role of local analgesic agents in decreasing post-operative pain and prolonging the duration of analgesia post-operatively has been applied in many researches. Nirmala and colleagues studied the role of a bupivacaine and buprenorphine combination and found that this combination was effective in reducing the total analgesic requirements and in increasing the duration of the post-operative analgesic time [10]. Another article reported the role of bupivacaine with morphine wound infiltration and concluded the same results [11], while Iqbal Singh and associates compared wound infiltration to the intercostal nerve block method using bupivacaine and supported our results in relation to the superiority of wound infiltration in decreasing the total analgesic requirements and post-operative pain, as well as prolonging the first analgesic demand [12]. Whereas these studies used the local analgesic agents on small wound incisions (peri-tubal incisions of percutaneous nephrolithotomy), other research confirmed the efficacy of nephrectomy wound continuous infiltration in improving post-operative pain, recovery period and reducing the cost of analgesic care [13, 14]. On the other hand, Nirmala *et al*. showed that ropivacaine, when used in an intercostal nerve block, gave superior analgesic efficacy to peri-tubal infiltration post percutaneous nephrolithotomy, as evidenced by the reduction in the amount of analgesic demands and consumption, and also in the longer time to first analgesic rescue [15].

In general, there is much literature studying the efficacy of wound infiltration as a method of post-operative analgesia, some supporting the role of wound infiltration alone in enhancing recovery period by decreasing the post-operative pain and the total analgesics demands [16], while others report the superiority of this method to placebo, but find that it is still inferior to the other gold standard analgesic techniques such as the epidural and the peripheral nerve block [17]. In terms of other major procedures, apart from in the urological field there are many studies that document the efficacy of continuous wound infiltration with local analgesic agents in lowering post-operative pain, analgesic requirements and recovery duration, such as after open gastrectomy, caesarean section, with equal efficacy to abdominal nerve blocks in caesarean sections and showing a possible reduction in the opioid requirements but not the pain scores post caesarean section [18], and pre-hysterectomy local analgesic wound infiltration, while this technique post-hysterectomy had no analgesic prolongation effect [19]. In the paediatric surgery field, a comparison of the effect of pre-incision nerve block to post-incision wound infiltration of the inguinal area during the herniotomy procedure found that the two methods provided the same adequate post-operative analgesia [20]. In total, the infiltration of the nephrectomy wound by combined local anaesthetic agents (0.5% Bupivacaine + 2% lidocaine) is more effective than an intercostal nerve block by same agents, as shown by the results of this study in relation to the post-operative pain and analgesic demands. In other aspects, the two methods of intra-operative nephrectomy wound analgesia did not have any superiority.

## Conclusion

The infiltration of flank wound incision and the intercostal nerve block post-operatively, with combined local analgesic agents (0.5% Bupivacaine + 2% lidocaine), are both effective techniques in improving the recovery period due to enhancing the post-operative pain, decreasing the total analgesic consumption during the first post-operative 72 hrs and prolonging the time to the first rescue analgesic demand, but the superiority for providing better outcomes (more pain control and reducing the cost of analgesic post-operative care) significantly favours the wound infiltration method. Thus, wound infiltration is superior to the intercostal nerve block in terms of the analgesic aspect and its use is recommended post-operatively in major urological procedures.

### What is known about this topic

- Post operative pain control due to open renal flank incision articles are scare.

### What this study adds

- This study demonstrates types of pain relieve in intercostal incisions used for kidney surgery.
